# Is an individually tailored programme of intense leg resistance and dynamic exercise acceptable to adults with an acute lateral patellar dislocation? A feasibility study

**DOI:** 10.1186/s40814-021-00932-x

**Published:** 2021-11-08

**Authors:** Colin Forde, Mark Haddad, Shashivadan P. Hirani, David J. Keene

**Affiliations:** 1 Nuffield Department of Orthopaedics, Rheumatology and Musculoskeletal Sciences, Kadoorie Research Centre, University of Oxford, Kadoorie Centre for Critical Care Research and Education, Level 3, John Radcliffe Hospital, Headley Way, Headington, Oxford, OX3 9DU UK; 2 School of Health Sciences, City, University of London, London, UK

**Keywords:** Physical therapy, Rehabilitation, Kneecap, Patellofemoral, Instability, Conservative, Non-operative, Pilot

## Abstract

**Background:**

Lateral patellar dislocations mainly affect active teenagers and young adults. To help people recover, non-surgical exercise-based treatment is often recommended but the optimal exercise-based treatment is unknown. Currently, treatment outcomes after this injury are variable. Common problems include recurrent dislocation, reduced activity levels, and later surgery. A programme of intense leg resistance exercises, and dynamic exercises related to participants’ activity-related goals, has rationale, but has not been previously reported. In line with the Medical Research Council guidance, this study aimed to assess the acceptability of a novel evidence-based exercise programme for adults after acute lateral patellar dislocation and the feasibility of future research evaluating this treatment.

**Methods:**

A single-group prospective study was conducted at the John Radcliffe Hospital, Oxford, UK. Participants were 16 years or older with an acute first-time or recurrent lateral patellar dislocation. Participants received up to six face-to-face, one-to-one, physiotherapy sessions, over a maximum of 3 months, and performed intensive home exercises independently at least three times per week. Strategies to increase exercise adherence were used. Primary objectives were to determine the number of eligible patients, the recruitment rate (proportion of eligible patients that provided written informed consent), participant adherence to scheduled physiotherapy sessions and self-reported adherence to prescribed exercise, and intervention acceptability to participants measured by attrition and a study-specific questionnaire. Data were analysed using descriptive statistics.

**Results:**

Fifteen of 22 (68%) patients with a lateral patellar dislocation were eligible. All eligible (100%) were recruited. Two of 15 (13%) participants provided no outcome data, 2/15 (13%) provided partial outcome data, and 11/15 (73%) provided all outcome data. Questionnaire responses demonstrated high intervention acceptability to participants. Participants attended 56/66 (85%) physiotherapy sessions and 10/11 (91%) participants reported they ‘always’ or ‘often’ completed the prescribed exercise. One participant redislocated their patella; another experienced knee pain or swelling lasting more than one week after home exercise on three occasions.

**Conclusion:**

The intervention appeared acceptable to adults after acute lateral patellar dislocation, and a future randomised pilot trial is feasible. This future pilot trial should estimate attrition with increased precision over a longer duration and assess participants’ willingness to be randomised to different treatments across multiple centres.

**Trial registration:**

ClinicalTrials.govNCT03798483, registered on January 10, 2019

**Supplementary Information:**

The online version contains supplementary material available at 10.1186/s40814-021-00932-x.

## Introduction

Patellar dislocations occur when the patella is forced out of the femoral trochlear groove, normally in a lateral direction. This is usually a non-contact injury [1] that mainly happens during sport [2]. The reported incidence of first-time patellar dislocations is 42/100,000 person years, equal between sexes, and highest in 10–17-year-olds [3]. After a first-time patellar dislocation, the average 10-year redislocation rate is 22.7%, with the highest rate being 35.5% which occurs in people aged 10–17 years at the time of injury [3].

Patellar dislocation treatment is either surgical or non-surgical [4]. Current evidence shows approximately two-thirds of patients treated non-surgically will not redislocate their patella at 6 to 9 years after injury and experience fewer complications and similar knee function and activity levels, compared to surgically treated patients [4]. Consequently, initial non-surgical treatment is recommended for most people after patellar dislocation and surgery reserved for those with large osteochondral fragments or who fail non-surgical treatment [5, 6]. Currently, non-surgical treatment outcomes are variable. Common problems include reduced activity levels [7, 8], later surgery [4], recurrent dislocation [9], and increased risk of symptomatic patellofemoral osteoarthritis [10].

The most effective non-surgical treatment is unknown due to a lack of high-quality randomised controlled trials (RCTs) [9]. Typically, non-surgical treatment involves brief immobilisation, advice, and physiotherapy prescription of leg flexibility, resistance, and proprioceptive exercises [9, 11]. Intense leg resistance and dynamic exercises, such as hopping and changing direction, are rarely used [9, 11]. However, this mainly young active patient population experiences most instability symptoms during multidirectional running and hopping activities [12]. Persistent deficits in knee extensor muscle strength have also been identified [13]. This indicates current rehabilitation programmes may be inadequate. A programme of leg resistance exercises prescribed in accordance with evidence-based guidelines [14], and dynamic exercises that prepare patients for the demands of the activities they wish to resume, could improve outcomes in terms of regaining function and reducing the risk of recurrence.

Whether a programme of structured resistance and dynamic exercises guided by a physiotherapist would be acceptable to patients and clinicians after acute lateral patellar dislocation (LPD) is unclear. Previous studies have reported this patient population’s attendance at physiotherapy is variable [15, 16]. The only published RCT that compared exercise-based interventions after acute patellar dislocation experienced 52% attrition [17], indicating loss to follow-up may make larger-scale multicentre research unfeasible. Investigating fidelity of treatment delivery is key as physiotherapists may find implementation challenging if the intervention is not consistent with their usual practice. Our study aimed to (1) provide preliminary evidence on the acceptability of a novel evidence-based intensive exercise-based intervention for adults after acute LPD and (2) assess the viability of a future multicentre pilot RCT. This programme of research follows the United Kingdom (UK) Medical Research Council’s guidance for developing and testing complex interventions, which recommends feasibility testing to address uncertainties in intervention and trial design before a full-sale evaluation [18]. Primary objectives were to determine the:

- Number of eligible patients
- Recruitment rate
- Intervention acceptability to participants
- Participant adherence to scheduled physiotherapy sessions and prescribed exercise

Secondary objectives were to:

- Assess the acceptability to participants of patient-reported outcome measures (PROMs) that could be used in a definitive trial
- Measure treatment-related adverse events
- Determine what assessment findings are reported by clinicians confirming a LPD diagnosis to inform eligibility criteria for future research
- Assess fidelity of intervention delivery to determine if the intervention can be delivered as intended in the UK National Health Service (NHS)

## Materials and methods

This single-group prospective feasibility study was conducted at the John Radcliffe Hospital, Oxford, a UK major trauma centre. The study protocol was prospectively registered and is available at ClinicalTrials.gov (NCT03798483). Ethical approval was granted by the Proportionate Review Sub-committee of the West of Scotland REC 5 (reference: 18/WS/0211). Reporting adheres to template for intervention description and replication (TIDieR) guidelines [19] and Consolidated Standards of Reporting Trials (CONSORT) extension to randomised pilot and feasibility trial guidelines [20].

### Eligibility criteria

Included participants were:

- Aged 16 years or older (reflecting the patients attending the adult trauma physiotherapy service at the study site)
- Attending a trauma clinic or referred to physiotherapy
- Had a first-time or recurrent LPD reduced by paramedics or diagnosed by an orthopaedic clinician

Exclusion criteria were:

- Anterior cruciate ligament or posterior cruciate ligament injury confirmed by a positive Lachman’s or posterior drawer test or magnetic resonance imaging
- Medial collateral ligament or lateral collateral ligament injury requiring hinged knee brace application or surgical repair
- Concomitant injury that would prohibit intervention participation
- More than 4 weeks from injury to Emergency Department or trauma clinic attendance
- Previous surgery on the affected knee
- Fracture(s) on plain radiograph including osteochondral fractures
- Medial patellar dislocation
- Considered inappropriate for physiotherapy by the assessing clinician
- History of severe neuromuscular or congenital disorders
- Listed for surgery prior to intervention completion
- Unable to attend physiotherapy appointments, understand written or spoken English, or give written informed consent

The local Emergency Department treatment pathway for patients with a suspected isolated LPD was application of a splint and referral to a consultant orthopaedic surgeon-led trauma clinic. Research nurses screened these clinic lists to identify potentially eligible patients. Potentially eligible patients were assessed by an orthopaedic clinician (surgeon, specialist nurse, or specialist physiotherapist) as per his/her usual practice. Diagnostic criteria were not specified. If a LPD was diagnosed, the same clinician who confirmed the diagnosis also assessed eligibility. Eligible patients were invited to discuss the study with a researcher and, if agreeable, provided written informed consent.

Research nurses were not available at weekends or bank holidays, so trauma clinic lists, as well as all physiotherapy referrals, were screened retrospectively. Potentially eligible patients identified this way were sent a participant information sheet and a letter inviting them to register their interest in participating by email or telephone. For those that were interested, a consultation to review eligibility and obtain informed consent was arranged.

### Intervention

Before intervention development, the existing evidence on non-surgical treatment for people with LPDs was reviewed, in keeping with the UK Medical Research Council guidance for complex intervention development [18]. Systematic review evidence has shown there is a lack of high-quality evidence to support one specific non-surgical treatment after patellar dislocation [9], and currently, there are no clinical guidelines to guide physiotherapy treatment after acute patellar dislocation. Therefore, we designed the intervention considering evidence related to the LPD mechanism of injury, patellofemoral biomechanics, common post-injury impairments, and approaches to support exercise adherence.

Most LPDs are thought to occur when changing direction with the knee relatively extended, and the femur internally rotated and adducted on an externally rotated tibia. This view is supported by studies which demonstrated increased lateral patellar displacement in early knee flexion [21] and with tibial external rotation [22]. Maintaining a relatively extended knee during dynamic activities, such as landing, could also increase redislocation risk as patients after LPD will usually have a medial patellofemoral ligament deficient knee [23] and this ligament provides most restraint to lateral patellar translation in early knee flexion [24]. Trunk position can also affect the direction and extent of knee joint moments by changing the location of the ground reaction force [25]. For example, ipsilateral trunk side flexion, which may result from hip abductor weakness, can create a valgus knee moment.

Strong hip and thigh muscles are thought to increase patellar stability by absorbing external hip and knee moments, preventing movement patterns associated with the LPD mechanism of injury [6]. In other patient populations, hip muscle weakness has been associated with knee valgus [26], and quadriceps weakness with reduced knee flexion on single leg landing [27, 28]. Higher quadriceps strength also protects against patellofemoral joint cartilage loss and is associated with less pain and higher physical function, in people with patellofemoral osteoarthritis [30].

Our intervention therefore aimed to use evidence-based exercise prescription to restore leg muscle strength and improve leg and trunk alignment during dynamic exercises related to participants’ activity-related goals. The rationale was that this would reduce instability symptoms and re-injury risk, restore pre-injury activity levels, and improve knee pain and function.

Six orthopaedic trauma physiotherapists (UK NHS bands 5–7) provided the intervention at the recruiting centre’s outpatient physiotherapy department. An initial iteration of the intervention, developed by the study team, was presented to physiotherapists who provided feedback. Feedback from physiotherapists resulted in alterations to the planned exercises (e.g., addition of supine inner range quadriceps exercise) and strategies to support participant adherence to prescribed exercise (e.g., removing requirement for physiotherapists and participants to sign action plan document). No formal process was used to reach consensus on intervention components. Before intervention administration, physiotherapists attended a -hour group training session that explained the study rationale, intervention, and procedures and involved exercise prescription practice.

**Fig. 1:**
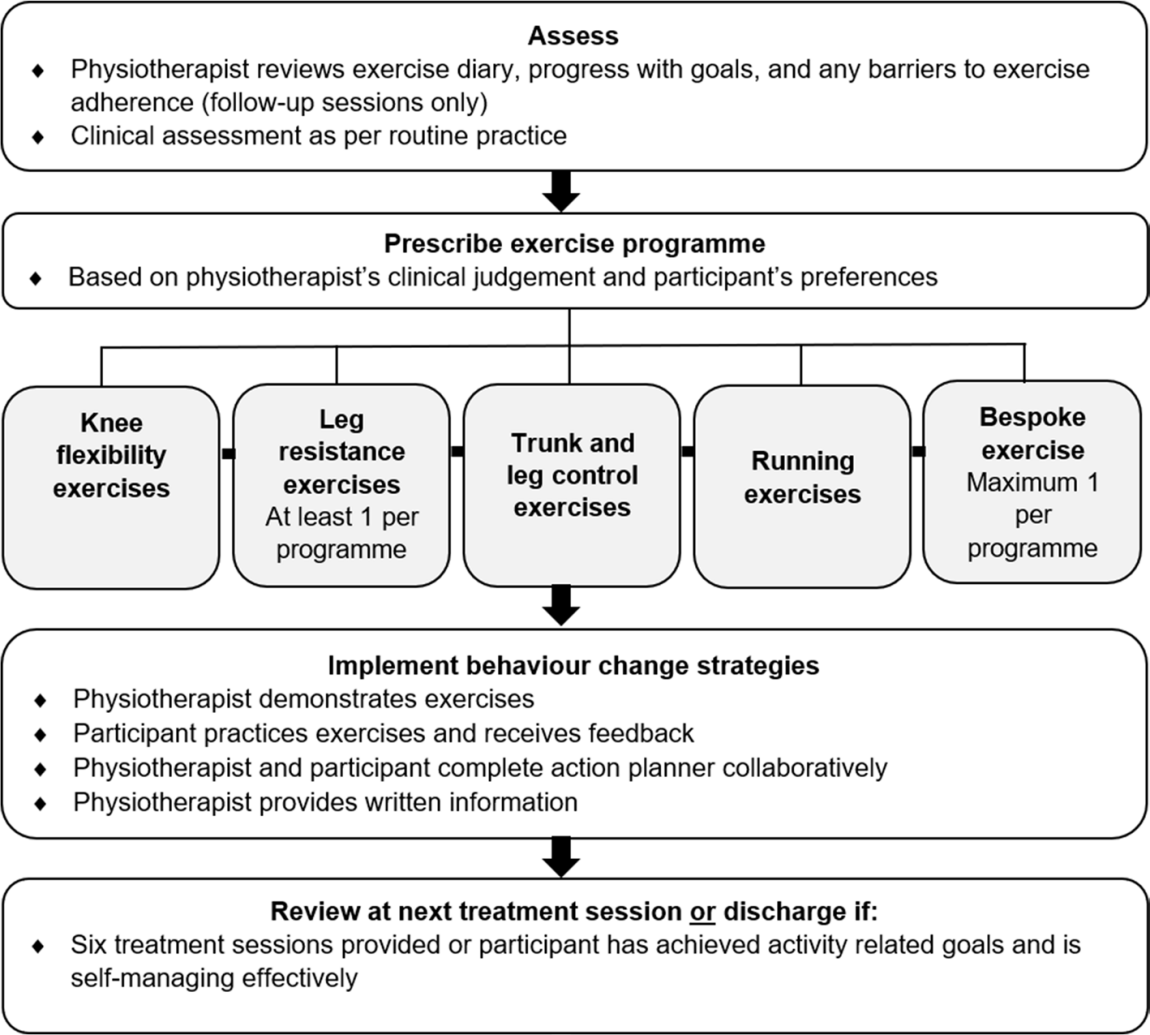
Overview of the intervention delivery process

Up to six, face-to-face, one-to-one sessions, over a maximum of 3 months were allowed. A maximum treatment duration of 3 months reflects reported practice by NHS physiotherapists for patients after first-time patellar dislocation [11]. Fewer than six sessions were used if participants achieved their goals and were self-managing effectively. Up to two additional sessions were allowed if clinically essential. Initial sessions were 45 min and follow-up sessions were 30 min. Session frequency was negotiated between physiotherapists and participants. An overview of the intervention delivery process is presented in Fig. 1.

Following routine clinical assessment, physiotherapists prescribed a maximum of five exercises to be performed by participants independently, based on their clinical judgement and participants’ preferences. Exercises were from a pre-determined list of knee flexibility, leg resistance, trunk and leg control, and running exercises of progressive difficulty. Physiotherapists could prescribe one bespoke exercise, not from this list, to help participants achieve their specific activity-related goals. A maximum of five exercises allowed one exercise to be chosen from each exercise category, if indicated. The restricted number of exercises also aimed to increase adherence; prescription of more than six exercises is associated with reduced patient adherence to prescribed home exercise [31]. At least one resistance exercise was prescribed per treatment session, in accordance with the American College of Sports Medicine guidelines [14], once pain allowed. During assessments, participants measured resistance exercise intensity by performing two repetitions and rating their perceived exertion from 0 to 10 [32]. The target intensity was 4–6, as five equates to 60–65% of one repetition max [33]. Participants then performed six more repetitions to ensure eight repetitions could be completed. The dose for flexibility, control, and running exercises was decided by physiotherapists as the optimal dose for these exercise types is less certain [34]. The intervention exercises and prescription instructions are available in Additional file 1.

To increase participant adherence to prescribed exercise, the following behaviour change strategies, derived from general NHS health trainer guidance [35], a consensually agreed taxonomy of techniques [36], and systematic review evidence [37, 38], were used: physiotherapist demonstration of prescribed exercise, participant practice of exercises with feedback, provision of written information (participant information booklet, exercise diary, and exercise sheets with pictures and instructions), and action planning (participants set an activity-related goal(s) to achieve on completion of treatment and by the next treatment session following Specific, Measurable, Achievable, Realistic, and Time-based principles; participants planned where and when to perform prescribed exercise;, and participants' confidence to adhere to the set programme was assessed). If confidence was low, or barriers to adherence were identified, these were problem solved by participants and physiotherapists collaboratively. Participants were asked to record any barriers to exercise adherence subsequently experienced and to bring exercise diaries and action planners to follow-up sessions. At follow-up sessions, physiotherapists reviewed exercise diaries and action planners and revised the prescribed exercise programme as described previously. Exercise diaries were used to facilitate, rather than assess, exercise adherence, so participants’ completion of exercise diaries was not recorded, nor was content analysed.

### Outcomes

Follow-up was three months after the first treatment session by postal questionnaire, unless a participant’s final treatment session was within one week of this time, in which case they completed follow-up questionnaires after their last session.

Primary outcomes were the:

- Number of eligible participants: proportion of patients diagnosed with a LPD that satisfied the eligibility criteria
- Recruitment rate: proportion of eligible patients who provided written informed consent
- Intervention acceptability to participants: measured by attrition (proportion of participants who did not provide follow-up data) and participant response to a study-specific questionnaire. As no established self-reported measure to assess intervention acceptability exists [39], we designed a questionnaire based on components of intervention acceptability [39] and an existing patient satisfaction questionnaire [40]. This measured satisfaction with treatment, self-efficacy, burden of treatment, and intention to adhere (see Table 2)
- Participant adherence: proportion of scheduled physiotherapy sessions attended and participant response at follow-up to the following: ‘how often did you perform your exercises at least three times a week’ and ‘when performing your home exercise programme, how often did you perform all of the exercises in your programme?’ These used 5-point Likert scales anchored at ‘always’ (zero) and ‘never’ (four)

Secondary outcomes were to assess:

- Acceptability of PROMs (completed at baseline after informed consent was obtained) that could be used in a definitive RCT by measuring the completion rates (proportion of questions in completed PROMs answered) of the:
  - ° Tegner Activity Scale, an activity scale from 0 to 10 (higher scores indicate higher activity levels) [41] with demonstrated reliability and validity in people with a patellar dislocation [42]. At baseline, pre-injury scores were used
  - ° Lysholm Knee Scoring Scale, an 8-item knee-specific scale scored from 0 to 100 (lower scores indicate higher disability) [41] with demonstrated reliability and validity in people with a patellar dislocation [42]. At baseline, current symptoms were used
  - ° EQ-5D-5L, which assesses the quality of life under five domains: mobility, self-care, usual activities, pain/discomfort, and anxiety/depression [43]. These are combined to give a score from −0.594 to 1 for UK populations (higher scores indicate higher quality of life). Participants also rate their health on a visual analogue scale from 0 to 100 (higher scores indicate better health). At baseline, the current health state was used
- Treatment-related adverse events: defined as any untoward sign or symptom related to completing the study intervention. These were monitored by physiotherapists at treatment sessions and by a follow-up questionnaire. Delayed onset muscle soreness, increased knee pain, and increased swelling lasting less than 1 week and not requiring medical attention were not considered adverse events
- What assessment findings are reported by clinicians confirming a LPD diagnosis: after diagnosing a LPD, orthopaedic clinicians were asked which of the following assessment findings we plan to use as eligibility criteria in a future study were present/absent/not assessed during their clinical assessment: convincing participant history of a visible deformity on the lateral aspect of the knee or sensation of the patella ‘popping’ out of joint followed by spontaneous reduction, a knee haemarthrosis or joint effusion, medial patellofemoral complex tenderness, and apprehension on lateral patellar displacement
- Intervention delivery, by analysing physiotherapist-completed treatment logs for initial injury management, duration from injury to the first treatment session, number of treatment sessions attended, physiotherapy treatment duration, prescribed exercises, and physiotherapists’ fidelity to implementing behaviour change strategies and prescribing resistance exercises as intended. Participants’ preferred intervention duration, number of physiotherapy sessions, and follow-up method (electronic, post, do not mind) were also assessed by a follow-up questionnaire

Telephone or email contact was used to encourage participants to complete follow-up and to obtain missing data where necessary. Due to the preliminary nature of this feasibility study, criteria to proceed to a definitive trial were not prespecified.

### Sample size

The sample size of 15 participants was pragmatic, based on previous local clinical data and the resources available. The 6-month recruitment period was based on an estimated 54 eligible patients and a 25% recruitment rate.

### Statistical methods

All data were analysed using descriptive statistics. Continuous and ordinal data were reported using medians and interquartile ranges. Categorical data were expressed as integers and percentages. Analyses were conducted using SPSS version 25 (IBM Corp. Armonk, NY) and Excel version 2007 (Microsoft Corp. Redmond, Washington). Combined EQ-5D-5L scores were calculated using the EQ-5D-3L crosswalk value set [44].

## Results

**Fig. 2:**
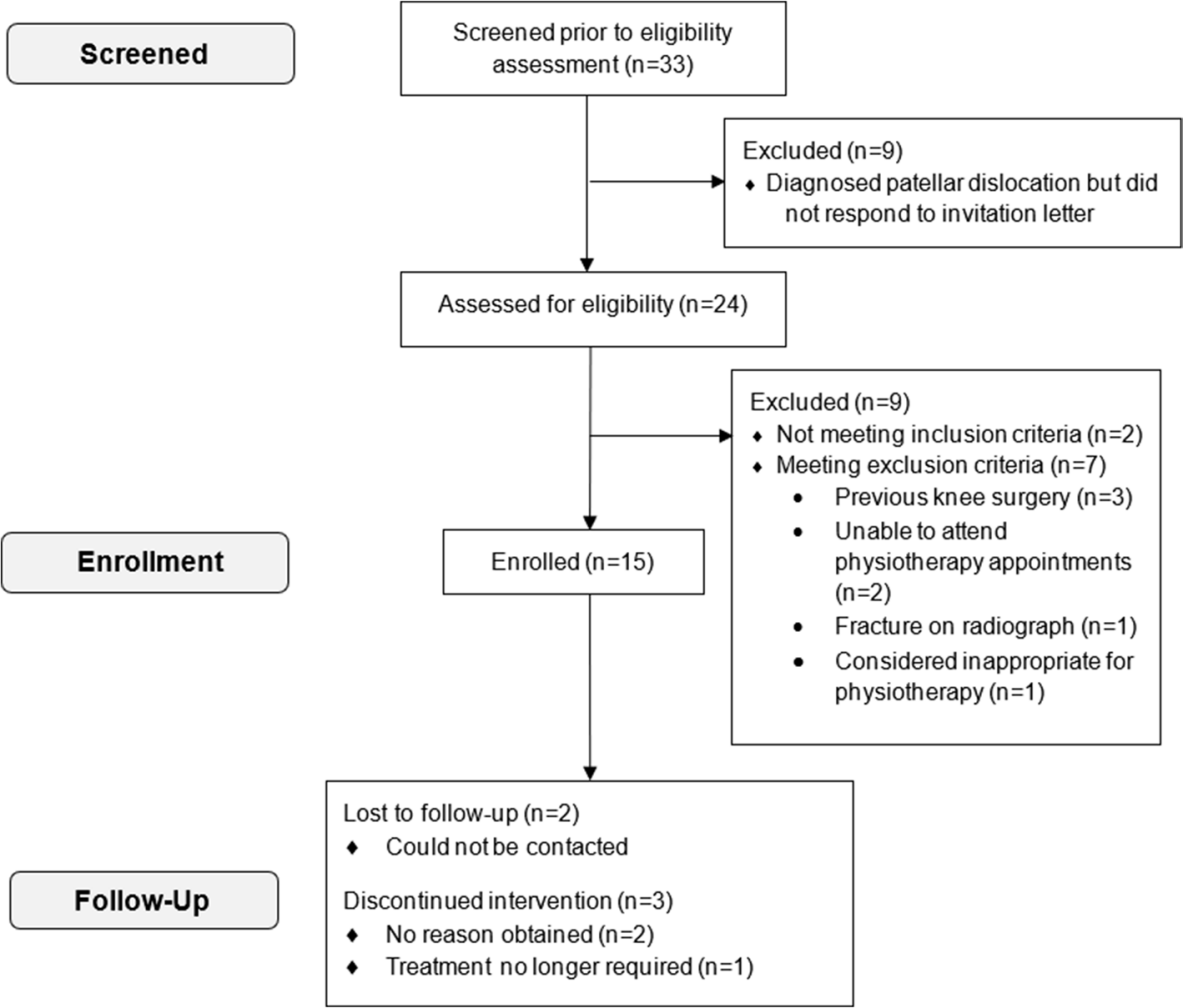
Flow of participants through the study

Recruitment started in January 2019 and finished in May 2019 when the recruitment target was reached. Follow-up was completed in October 2019. The flow of participants through the study is presented in Fig. 2. In total, 33 potentially eligible patients were identified. Ten patients with a diagnosed patellar dislocation did not undergo eligibility assessment at a trauma clinic. These ten patients were sent a study invitation pack. One responded and subsequently underwent an eligibility assessment. So, 24 patients were assessed for eligibility and 22 were diagnosed with a LPD.

### Primary outcomes

Fifteen of 22 (68.2%) patients diagnosed with a LPD satisfied the eligibility criteria. All eligible patients consented to participate (3.9 participants recruited per month). Baseline demographics and clinical characteristics of participants are presented in Table 1.

**Table 1:** Baseline demographics and clinical characteristics

Number of participants	15
Age (years)	22 (19–28)
Sex (female)	7 (46.7%)
Duration from injury to eligibility assessment (days)	2 (1–9)
Ipsilateral patellar dislocation before current dislocation (yes)	5 (33.3%)
Number of ipsilateral patellar dislocations before current patellar dislocation (number of participants)	
1	1 (6.7%)
2	1 (6.7%)
3	1 (6.7%)
4	1 (6.7%)
5–6	1 (6.7%)
Previous contralateral patellar dislocation (yes)	3 (20%)
Number of previous contralateral patellar dislocations (number of participants)	
1	1 (6.7%)
2	1 (6.7%)
>10	1 (6.7%)
Family history of patellar dislocation (yes)	1 (6.7%)
Height (metres)	1.75 (1.62–1.8)
Weight (kg)	69.9 (64–85)
Ethnicity (number of participants)	
White British	13 (86.7%)
White Other	1 (6.7%)
Other	1 (6.7%)
Education (number of participants)	
Secondary education	9 (60%)
Higher professional or university education	6 (40%)
Employment (number of participants)	
Employed	12 (80%)
Student	3 (20%)

Data are median (interquartile range) unless otherwise stated; *kg* kilogrammes

Attrition was 13%, with two participants not providing any follow-up data. Follow-up was by telephone for two participants as they had not returned follow-up questionnaires several weeks after the 3-month follow-up time point despite email and telephone reminders. Only Lysholm Knee Scoring Scale outcome data was obtained from these participants; this was the only knee-specific PROM and was therefore prioritised. Eleven of 15 (73.3%) participants completed our study-specific intervention acceptability questionnaire. Responses are summarised in Table 2, overall indicating a positive experience of the intervention.

**Table 2:** Intervention acceptability participant questionnaire (*n* = 11)

How satisfied are you with the effect of your physiotherapy treatment? (very satisfied–very dissatisfied)	0 (0–0)
How satisfied are you with your involvement in decision-making about your physiotherapy treatment? (very satisfied–very dissatisfied)	0 (0–0)
This study offered up to six physiotherapy sessions over 3 months after your injury, how satisfied were you with this amount of treatment? (very satisfied–very dissatisfied)	0 (0–0)
How satisfied were you with the written information you were given describing the study? (very satisfied–very dissatisfied)	0 (0–0)
How satisfied were you with the written information you were given about your injury? (very satisfied–very dissatisfied)	0 (0–1)
How satisfied are you overall with the physiotherapy care you received after your injury? (very satisfied–very dissatisfied)	0 (0–0)
How confident are you that you can return to all your normal activities? (very confident–not at all confident)	0 (0–1)
How did doing your exercises fit into your weekly routine? (very easy–very difficult)	1 (0–2)
How confident are you that you were doing your exercises the way your physiotherapist showed you? (very confident–not at all confident)	1 (0–1)
How confident are you that you understood how tiring the muscle strengthening exercises should feel? (very confident–not at all confident)	1 (0–1)
How likely are you to continue your exercises now your physiotherapy is finished? (very likely–very unlikely)	1 (0–1)

Data are median (interquartile range); answers to questions were on a 5-point Likert scale (0–4), and the Likert scale anchors are presented in brackets after questions

Participants attended 56/66 (84.8%) scheduled physiotherapy sessions. In response to ‘when performing your home exercise programme, how often did you perform all of the exercises in your programme?’, 4/11 participants (36.4%) reported ‘always’, 5/11 (45.5%) reported ‘often’, and 2/11 (18.2%) reported ‘sometimes’. In response to ‘how often did you perform your exercises at least three times a week?’, 5/11 (45.5%) reported ‘always’, 5/11 (45.5%) reported ‘often’, and 1/11 (9.1%) reported ‘sometimes’.

### Secondary outcomes

There were no missing data from PROMs completed at baseline and returned at follow-up. PROM scores are presented in Table 3. Participants reported no treatment-related adverse events while attending physiotherapy. At follow-up, one of the eleven participants who provided data reported knee pain or swelling after completing home exercise lasting more than 1 week, on three occasions. One participant who completed treatment but did not complete follow-up was later reported to have experienced a recurrent LPD by a clinician from another hospital. Attempts to contact this participant to determine the cause of this dislocation were unsuccessful.

**Table 3:** Patient-reported outcome measure scores

	Baseline (*n* = 15)	Follow-up (*n* = 11)
Tegner Activity Scale	6 (4–7)	6 (3–7)
Lysholm Knee Scoring Scale	44 (34–55)	^a^90 (76.5–95)
EQ-5D-5L		
Combined score	0.56 (0.49–0.69)	0.84 (0.8–1.0)
Visual analogue scale	65 (40–90)	90 (85–95)

Data are median (interquartile range); *n*, number of participants. ^a^Follow-up data from 13 participants

On routine assessment, orthopaedic clinicians reported 15/15 (100%) participants had medial patellofemoral complex tenderness, 13/13 (100%) participants had apprehension on lateral patellar displacement, 14/15 (93.3%) participants had a convincing history of LPD, and 10/15 (66.7%) participants had a knee joint haemarthrosis or effusion. Patellar apprehension was not assessed on two occasions.

Delivery of the study intervention is summarised in Table 4. The median number of physiotherapy sessions attended was three (*IQR* 3–5) and treatment duration was 50 (37–79) days. Leg resistance exercises were most frequently prescribed during treatment sessions (50/55 sessions), followed by trunk and leg control (45/55 sessions), knee flexibility (27/55 sessions), bespoke (12/55), and running (11/55 sessions) exercises. The frequency that individual exercises were prescribed is available in Additional file 2.

**Table 4:** Delivery of the study intervention

**Duration from injury to first treatment session (days)**	21 (15–27)
**Prior to the first treatment session**	
Immobilisation (yes)	
Lateral buttress splint	12 (80%)
Cricket pad splint	2 (13.3%)
Hinged knee brace	1 (6.7%)
Weight-bearing status	
Full	15 (100%)
Walking aids	
None	8 (53.3%)
Two elbow crutches	7 (46.7%)
Exercises prescribed	
Knee range of movement exercise	7 (46.7%)
Non-weight-bearing knee strengthening exercises	4 (26.7%)
Gait practice, balance exercises	2 (13.3%)
Weight-bearing knee strengthening, strengthening of uninjured joints	1 (6.7%)
**Physiotherapy treatment**	
Physiotherapy sessions (total)	55
Physiotherapy sessions (median)	3 (3–5)
Physiotherapy duration (days)	50 (37–79)
Number of participants prescribed exercise	
Knee flexibility	13 (86.7%)
Trunk and leg control	14 (93.3%)
Leg resistance	15 (100%)
Running	5 (33.3%)
Bespoke	5 (33.3%)
Number of sessions where exercise prescribed	
Knee flexibility	27 (49.1%)
Trunk and leg control	45 (81.8%)
Leg resistance	50 (90.9%)
Running	11 (20%)
Bespoke	12 (21.8%)

Data are median (interquartile range) unless otherwise stated; *IQR* interquartile range

Physiotherapist fidelity to implementing behaviour change strategies and resistance exercise prescription instructions are presented in Table 5. Fidelity to implementing behaviour change strategies and prescribing resistance exercise as intended was high, except for resistance exercise intensity.

**Table 5:** Physiotherapist fidelity to intervention delivery

^**a**^**Behaviour change strategies**	
**Action planner completed/reviewed**	
Yes	50/55 (90.9%)
No	5/55 (9.1%)
Participant did not bring to session	3/55 (5.5%)
Participant discharged/last treatment session	2/55 (3.6%)
^**b**^**Participant information booklet given**	
Yes	14/15 (93.3%)
No	^c^1/15 (6.7%)
None available	1/15 (6.7%)
**Exercise diary issued/reviewed**	
Yes	51/55 (92.7%)
No	4/51 (7.8%)
Participant did not bring to session	3/55 (5.5%)
Participant discharged/last treatment session	1/55 (1.8%)
**Exercise(s) demonstrated to participant?**	
Yes	50/55 (90.9%)
No	5/55 (9.1%)
Participant already completing exercises	3/55 (5.5%)
Participant discharged/last treatment session	2/55 (3.6%)
**Participant opportunity to practice exercise(s)**	
Yes	52/55 (94.5%)
No	3/55 (5.5%)
Participant already completing exercises	1/55 (1.8%)
Participant discharged/last treatment session	2/55 (3.6%)
^**d**^**Resistance exercise prescription**	
**≥1 resistance exercise prescribed per treatment session**	
Yes	50/55 (90.9%)
No	5/55 (9.1%)
No reason provided	3/55 (5.5%)
No exercises prescribed as participant discharged	2/55 (3.6%)
**Sets between 1 and 3**	
Yes	90/93 (96.8%)
No	3/93 (3.2%)
Missing data	2/93 (2.2%)
>3 sets prescribed	1/93 (1.1%)
**Reps between 8 and 12**	
Yes	89/93 (84.9%)
No	4/93 (4.3%)
Missing data	2/93 (2.2%)
>12 reps prescribed	1/93 (1.1%)
<8 reps prescribed	1/93 (1.1%)
**Frequency ≥3 times per week**	
Yes	91/93 (97.8%)
No	2/93 (2.2%)
Missing data	1/93 (1.1%)
<3/week	1/93 (1.1%)
**Intensity 4–6 after two repetitions**	
Yes	61/93 (65.6%)
No	32/93 (34.4%)
Insufficient weights available to reach target intensity	7/93 (8.4%)
Missing data	4/93 (4.3%)
Patient independent with this	1/93 (1.1%)
Squat exercise used as control exercise therefore intensity not regulated	1/93 (1.1%)
Completed before not reassessed	1/93(1.1%)
<4	12/93 (12.9%)
>6	6/93 (6.5%)

^a^The denominator for behaviour change strategies is the total number of physiotherapy sessions provided, that is 55. ^b^Participant information booklets were issued once; therefore, the total number equals the number of participants, that is 15. ^c^Subsequently issued to this participant at the second treatment session. ^d^The denominator for resistance exercises prescribed is the total number of resistance exercises prescribed by physiotherapists, that is 93

At follow-up, participants (*n* = 11) reported a preference for a median of six (6–7.25) physiotherapy sessions over a median of four (3–4.9) months. If participating in a future study, five participants (45.5%) would prefer electronic follow-up, five (45.5%) had no preference, and one (9.1%) would prefer postal follow-up.

### Ancillary analyses

The median follow-up timepoint was 15 (13–19) weeks. Two participants had treatment durations of 102 and 116 days, exceeding the 3 months permissible. No participant attended more than six sessions. Twelve participants completed treatment and three did not: two participants did not attend and did not re-schedule, and one participant cancelled a treatment session and later informed the lead author no further treatment was required.

## Discussion

To our knowledge, this is the first study to implement and describe an individually tailored programme of intense leg resistance and dynamic exercise using evidence-based prescription guidelines for adults after acute LPD. The findings indicate that the intervention was acceptable to participants and a multicentre pilot RCT assessing the feasibility of a definitive trial evaluating this intervention is viable.

Compared to other UK studies of non-surgical treatment after acute patellar dislocation, the eligibility rate of 68.2% is greater than Armstrong et al. [15] (19.5%) but less than Smith et al. [17] (89.3%). To reflect normal clinical practice at the participating centre, LPDs were diagnosed if reduced by paramedics or following orthopaedic clinician assessment. On assessment, most participants had medial patellofemoral complex tenderness, patellar apprehension, a knee haemarthrosis or effusion, and a convincing history of LPD. This indicates that these clinical findings, regularly used as LPD diagnostic criteria in other studies [15, 17, 44, 45], could be used to form eligibility criteria in future research to recruit a clinically representative sample

All eligible patients were recruited indicating the study intervention and procedures were prospectively acceptable to participants. We recruited 3.9 participants per month, more than previous studies of non-surgical treatment after acute patellar dislocation [15, 17, 46]. However, this was a single-centre study with research nurse support for patient screening and recruitment. Similar recruitment levels may not be achieved in centres without this level of support. A multicentre pilot RCT would provide a better estimate of recruitment for a definitive trial evaluating this intervention.

The low attrition and positive responses to our study-specific questionnaire indicate the study intervention and procedures were acceptable to participants, but we acknowledge reliability and validity of our questionnaire has not been established. Thirteen per cent attrition at 3 months compares favourably with 26% attrition at 6 weeks in the only RCT that compared exercise-based interventions after patellar dislocation [17]. As attrition is the main uncertainty of a future definitive RCT, a pilot study with longer follow-up and sample size to estimate attrition with increased precision is required. This pilot study should consider offering electronic follow-up as some participants reported this as their preferred follow-up method, and introducing an electronic follow-up option was associated with increased follow-up rates in a feasibility study comparing surgical and non-surgical treatment for recurrent patellar dislocation [16].

Participant adherence to scheduled physiotherapy sessions and prescribed exercise was high. Likert scales were used to measure participants’ exercise adherence as the optimal method for assessing self-reported adherence to prescribed exercise has not been established [47, 48]. However, the findings suggest the evidence-based behaviour change strategies used to increase exercise adherence may have been effective. It is unclear if the intervention restored leg strength and improved trunk and leg kinematics as intended, as we were unable to perform objective testing due to resource limitations.

Generally, there was high physiotherapist fidelity to implementing behaviour change strategies and prescribing resistance exercises as intended, demonstrating these intervention components are deliverable. There were some issues with regulating resistance exercise intensity with 34.4% of resistance exercises not prescribed at the target intensity (4–6 on the modified Borg scale). This intensity was potentially too high—other studies have used a starting intensity of 3–4 [49, 50]—for some participants in early-stage rehabilitation where pain is likely to be a limiting factor. This could explain why 12.9% of resistance exercises were prescribed at lower than the target intensity. However, 6.5% of resistance exercises were prescribed at a higher intensity and intensity could not be regulated for 8.4% of exercises due to insufficient weights at the study centre. These findings indicate a wider intensity range may be needed for resistance exercises to cater for the variable symptoms experienced during rehabilitation and the individual abilities of participants.

Running exercises were prescribed for 5/15 (33.3%) participants. This could be considered low as the median pre-injury Tegner score was six (*IQR* 4–7), which corresponds to recreational tennis and basketball [41]. However, three participants did not complete treatment and were not prescribed any running exercise which is understandable as running is typically a late-stage rehabilitation exercise; if these participants completed treatment, more running exercises may have been prescribed. It is also possible some participants chose not to return to sport due to changing priorities and the implications of re-injury, as seen in some patients after anterior cruciate ligament reconstruction [51]. Future intervention iterations should allow a longer treatment duration as two participants exceeded the maximum of 3 months and participants reported treatment lasting 4 months would be preferable. Based on informal physiotherapist feedback, a longer treatment duration would also facilitate running exercise prescription.

There was no missing data from completed PROMs indicating these were acceptable to participants. As no agreed outcome set exists for this patient group [9], we used PROMs to assess knee function, activity levels, and quality of life, as recommended [42]. Recently, the Norwich Patellar Instability (NPI) score (19 questions) [52] and Banff Patella Instability Instrument (BPII) 2.0 (23 questions) [53] have been developed to assess instability symptoms and quality of life, respectively, in patients with patellar instability. We did not use these as attrition can be an issue in this patient population and participants might consider these PROMs burdensome.

Treatment-related adverse events were rare: one participant reported expected complications of rehabilitation and there was one recurrent dislocation but it could not be established if this related to study participation. A redislocation rate of 6.7% (1/15) over 15 weeks is similar to studies of non-surgical treatment after first-time dislocations [17, 46]. Given five participants had recurrent dislocations, the study intervention appears safe though longer follow-up would be required to confirm this.

Until high-quality RCTs evaluating exercise-based treatments are conducted, theory can help inform the design and delivery of rehabilitation programmes for patients after LPD. The study intervention was designed following a review of the existing evidence for non-surgical treatment after LPD and refined following clinician feedback. It targets modifiable impairments—leg strength, and trunk and leg kinematics—that may predispose to poor outcome after LPD, can be tailored to patients’ individual needs, and uses strategies to support exercise adherence.

### Limitations

This was a single-centre study with a small sample size, so caution is required when making inferences based on our findings. Reflecting physiotherapy provision at the recruiting site, only people aged ≥16 years old were recruited; however, the incidence of first-time patellar dislocations is highest in 10–17-year-olds [3]. Due to resource limitations, we did not conduct qualitative research which could have helped us understand acceptability of the study intervention and procedures from participants’ perspectives, why some participants did not complete treatment or study follow-up, and why prescribing resistance exercises at the recommended intensity was problematic. This preliminary feasibility study was not developed or conducted with a patient and public involvement (PPI) representative or group. This would have provided a valuable perspective during the design of the intervention and when developing study processes and materials. Finally, this was a single-group study, so participants’ willingness to be randomised to a less intense treatment arm versus the study intervention is unknown.

## Conclusion

The intervention was acceptable to adults after acute LPD, and a multicentre pilot RCT assessing the feasibility of a definitive trial evaluating this intervention is viable. We have applied for and secured funding for this pilot RCT. Based on findings from this study, this pilot RCT will assess participants’ willingness to be randomised to a less intense treatment, assess recruitment across multiple centres, estimate attrition with increased precision over a longer duration, and conduct qualitative research to understand how the intervention is implemented and participants’ experience of study participation. We will also include paediatric participants. We have formed a PPI group who helped develop the design for the pilot RCT and will be involved through all stages of the project.

## Supplementary Information

**Additional file 1.** Intervention exercises and prescription instructions. The exercises prescribed as part of the study intervention and the prescription instruction for intervention providers.

**Additional file 2.** Prescribed exercises. The frequency that individual exercises were prescribed by intervention providers.

## Data Availability

The datasets used and/or analysed during the current study are available from the corresponding author on reasonable request.

## Abbreviations

CONSORT: Consolidated Standards of Reporting Trials
LPD: Lateral patellar dislocation
NHS: National Health Service
PPI: Patient and public involvement
PROM: Patient-reported outcome measure
RCT: Randomised controlled trial
TIDieR: Template for intervention description and replication
UK: United Kingdom
