# Supplementary material for: Is an individually tailored programme of intense leg resistance and dynamic exercise acceptable to adults with an acute lateral patellar dislocation? A feasibility study
Source: Pilot Feasibility Stud. 2021 Nov 8;7:197. doi: 10.1186/s40814-021-00932-x (PMC8573884; doi:10.1186/s40814-021-00932-x)
Supplement: Supplementary file 1 — Additional file 1. Intervention exercises and prescription instructions. The exercises prescribed as part of the study intervention and the prescription instruction for intervention providers. [file 40814_2021_932_MOESM1_ESM.docx]

| Intervention exercises and prescription instructions | | |
| --- | --- | --- |
| **Category** | **Exercise** | **Dose** |
| **Knee flexibility** | Sitting on chair AROM knee flexion | **Frequency:** ≥3/weeks  The remaining parameters of exercise dose were at physiotherapists’ discretion |
|  | Supine AROM knee flexion |  |
|  | Long sitting AAROM knee flexion |  |
|  | Kneeling bum to heels |  |
|  | Supine static quadriceps contraction |  |
|  | Supine static quadriceps contraction foot elevated |  |
|  | Sitting on chair knee extension using hands |  |
|  | Prone knee extension |  |
| **Trunk and leg control** | Weight shifting in standing | **Frequency:** ≥3/weeks  The remaining parameters of exercise dose were at physiotherapists’ discretion |
|  | Single leg stand with support |  |
|  | Single leg stand unsupported |  |
|  | Single leg squat with support |  |
|  | Single leg squat unsupported |  |
|  | Step forward and hold in single leg squat position |  |
|  | Double leg hop forward |  |
|  | Single leg hop forward unaffected to affected leg |  |
|  | Single leg hop forward affected to affected leg |  |
|  | Single leg hop forward over object affected to affected leg |  |
|  | Step laterally and hold in single leg squat position |  |
|  | Double leg hop laterally |  |
|  | Single leg hop laterally unaffected to affected leg |  |
|  | Single leg hop laterally affected to affected leg |  |
|  | Single leg hop laterally over object affected to affected leg |  |
| **Leg resistance exercises** | Supine inner range quadriceps unresisted | **Sets:** 1-3  **Reps:** 8-12  **Rest:** ≥2 minutes between sets  **Frequency:** ≥3/weeks  **Intensity:** 4-6 on modified Borg scale (0-10) |
|  | Seated knee extension with resistance band |  |
|  | Split squat with rear leg bias |  |
|  | Squat |  |
|  | Leg press |  |
|  | Rear foot elevated squat |  |
|  | Step-up |  |
|  | Single leg stiff-leg deadlift with support |  |
|  | Single leg stiff-leg deadlift unsupported |  |
|  | Isometric external rotation in semi-squat with resistance band |  |
| **Running**  **exercises** | Straight line running | **Frequency:** ≥3/weeks  The remaining parameters of exercise dose were at physiotherapists’ discretion |
|  | Deceleration |  |
|  | 45° cut |  |
|  | 90° cut |  |
|  | 180° turn |  |
| **Bespoke exercise** | Exercise prescribed by physiotherapists to facilitate participant goal attainment | **Frequency:** ≥3/weeks  The remaining parameters of exercise dose were at physiotherapists’ discretion |
| AROM, Active range of movement; AAROM, Active assisted range of movement | | |
