# Supplementary material for: Is an individually tailored programme of intense leg resistance and dynamic exercise acceptable to adults with an acute lateral patellar dislocation? A feasibility study
Source: Pilot Feasibility Stud. 2021 Nov 8;7:197. doi: 10.1186/s40814-021-00932-x (PMC8573884; doi:10.1186/s40814-021-00932-x)
Supplement: Supplementary file 2 — Additional file 2. Prescribed exercises. The frequency that individual exercises were prescribed by intervention providers. [file 40814_2021_932_MOESM2_ESM.docx]

| Prescribed exercises |  |
| --- | --- |
| **Total** | 215 |
| **Knee flexibility exercises** | 32 (14.9%) |
| Long sitting AAROM knee flexion | 12 (5.6%) |
| Kneeling bum to heels | 9 (4.2%) |
| Supine static quadriceps contraction, supine static quadriceps contraction foot elevated | 3 (1.4%) |
| Supine AROM knee flexion | 2 (0.9%) |
| Sitting on chair AROM knee flexion, sitting on chair knee extension using hands, prone knee extension | 1 (0.5%) |
| **Trunk and leg control exercises** | 63 (29.3%) |
| Single leg stand unsupported | 11 (5.1%) |
| Single leg squat unsupported | 9 (4.2%) |
| Single leg hop forward affected to affected leg, single leg hop laterally affected to affected leg | 8 (3.7%) |
| Single leg squat with support | 7 (3.3%) |
| Step forward and hold in single leg squat position | 6 (2.8%) |
| Single leg stand with support | 5 (2.3%) |
| Single leg hop forward unaffected to affected leg, step laterally and hold in single leg squat position, single leg hop laterally unaffected to affected leg | 2 (0.9%) |
| Double leg hop forward, single leg hop forward over object affected to affected leg, double leg hop laterally | 1 (0.5%) |
| Weight shifting in standing, single leg hop laterally over object affected to affected leg | 0 (0%) |
| **Leg resistance exercises** | 93 (43.3%) |
| Squat | 20 (9.3%) |
| Seated knee extension with resistance band | 15 (7%) |
| Supine inner range quadriceps unresisted, step-up | 12 (5.6%) |
| Leg press | 8 (3.7%) |
| Rear foot elevated squat, isometric external rotation in semi-squat with resistance band, single leg stiff-leg deadlift unsupported | 7 (3.3%) |
| Split squat with rear leg bias | 3 (1.4%) |
| Single leg stiff-leg deadlift with support | 2 (0.9%) |
| **Running exercises** | 15 (7%) |
| 90° cut | 5 (2.3%) |
| Straight line running, deceleration | 4 (1.9%) |
| 45° cut, 180° turn | 1 (0.5%) |
| **Bespoke exercises** | 12 (5.6%) |
| Seated knee extension machine | 4 (1.9%) |
| Sit-to-stand with affected leg behind | 3 (1.4%) |
| Static cycling, hamstring curls, inner range quadriceps in standing using resistance band, bouncing on trampette, jogging on trampette | 1 (0.5%) |
| AROM, Active range of movement; AAROM, Active assisted range of movement | |
